# The Road Murder Psychologically Considered

**Published:** 1861-07

**Authors:** 


### ART. IX.—THE ROAD MURDER PSYCHOLOGICALLY CONSIDERED.

"The seventeenth and eighteenth centuries, together with so much of the nineteenth as we have yet seen, jointly compose the Augustan age of murder."—*De Quincey.*

MURDER is a subject which exercises a terrible fascination over us. Blink the question as we may, we are conscious of an overweening interest in all that relates to the horrible act, even in its most common-place guise, whenever brought fairly home to the attention. Doubtless, under ordinary circumstances, while we munch our toast and sip our coffee, glancing meanwhile over the columns of the morning paper, the eye rests heedlessly upon the commoner examples of murderous brutality it too often finds there, and the attention is fully aroused only when arrested by the diabolical workmanship of a Youngman or a Mullins. But

* *The Great Crime of* 1860: being a Summary of the Facts relating to the Murder committed at Road; a Critical Review of its Social and Scientific Aspects; and an authorized account of the Family; with an Appendix containing the Evidence taken at the various Inquiries. By J. W. Stapleton, Surgeon, Trowbridge, Wilts, London: 1861, pp. 379.

let the ghastly deed shoulder us, as it were, it matters not in how trite a fashion—let the battered, hacked, or bullet-probed body of a vagrant or cadger, killed, perhaps, in a drunken brawl, be found in the ditch that bounds our demesne, or in a by-way that skirts our hamlet or little town, or within the margin of the wood that forms so picturesque an item of our every-day prospect, and whose shady recesses and wild paths form our best loved lounges, or in a nook or alley of the street we live in, or in the back-slums over which we gaze from the rear of our house in city or town—let murder thus shoulder us, and not even the absorbing whirl of active life will save us from the thrill of horror which follows from the near contemplation of the dire deed, and from the acute interest which surges up in our souls and eggs us on to learn every detail of the detested action.

How strange it is, and seemingly how paradoxical, that death in its first-begotten and most revolting aspect, should charm us to its contemplation, and evoke feelings which, if not positively pleasurable in themselves, yet give rise to a certain reflex of emotion from which we derive an undoubted but peculiar gratification. Let us not too hastily exclaim that this view is derogatory to humanity. We are here dealing with facts rather than opinions. The intimate relationship between antithetical emotions is a fact of no new date. The Executioners of the Law had, on the morning of the fatal day, removed the chains which restrained Socrates, and he sitting upon the edge of his bed, and rubbing his cramped leg, smilingly observed to the friends who now stood around him: "How strange a thing is that, my friends, which is called pleasure; and how oddly is it connected with its supposed opposite, pain. Pleasure and pain do not come to a man together, but if a person runs after the one and catches it, he almost invariably catches the other too, as if they were fastened together at one end. I think if Œsop had noticed this, he would have composed a fable to this effect: that the gods tried to reconcile these two opposites, and not being able to do this, fastened their extremities together; so that when you take hold of one, it pulls after it the other. And as it happens to me now, there was pain in my leg when the chain bound it, and now comes pleasure following the pain."* And Phædo thus describes the emotions which affected him on witnessing the judicial murder of the great philosopher:—

"I experienced peculiar emotions on that occasion. I did not feel compassion, as one might have expected I should, on being present at the death of a dear friend. I assure you, Echestratus, he appeared to me happy, both from his behaviour and from his discourse, with so much calmness and magnanimity did he meet death. I felt persuaded

---

* *Phœdo,* Whewell's trans.

that he quitted this life under divine protection; and that in another world he must be happy, if any one ever was. On this account I had no painful feeling of pity as might seem natural to a person present at such a catastrophe, nor did I feel pleasure, as on ordinary occasions when we were talking philosophy; though the discourse was of the same kind. It was a peculiar feeling which possessed me: a strange mixture of pleasure and grief, when I thought that he would soon cease to be. And we were all in this same mood—sometimes laughing, sometimes weeping; especially Apollodorus: he wept violently. You know the man and his way."*

We speak, therefore, rightly of the luxury of grief,† and with equal justness of the gratefulness of terror.‡ From this close linking together of painful and pleasurable emotions, Sir W. Hamilton conceives a strong confirmation is derived of the doctrine "that all pleasure is a reflex of activity, and that the free energy of every power is pleasurable;"§ and he argues (very aptly for the point we have in question) that on the same principle is to be explained the enjoyment which men have in spectacles of suffering—in the combats of animals and men, in executions, tragedies, and so forth, a disposition which, as he observes, "not unfrequently becomes an irresistible habit, not only for individuals, but for nations." He further adds:—"The excitation of energetic emotions painful in themselves is, however, also pleasurable. St. Austin affords curious examples of this in his own case, and in that of his friend Alypius. Speaking of himself in his *Confessions*, he says:—'Theatrical spectacles were to me irresistible, replete as they were with images of my own miseries, and the fuel of my own fire. What is the cause why a man chooses to grieve at scenes of tragic suffering, which he would have the utmost aversion himself to endure? And yet the spectator wishes to derive grief from these; in fact, the grief itself constitutes his pleasure. For he is attracted to the theatre, not to succour, but only to condole."

---

* *Phædo*, Whewell's trans.

† — "Mourning and grieving for all, ofttimes sitting in my palace, sometimes again I am delighted in my mind with grief."—*Odyssey*, b. iv. l. 100.

"*K. Philip.* You are as fond of grief as of your child.
"*Constance.* Grief fills the room up of my absent child,
Lies in his bed, walks up and down with me;
Puts on his pretty looks, repeats his words,
Remembers me of all his gracious parts,
Stuffs out his vacant garments with his form;
Then have I reason to be fond of grief."—*King John*, act iii. scene 3.

‡ "Each trembling heart with grateful terrors quell'd."—*Akenside.*

§ *Metaphysics*, lect. xliv. More fully:—"We feel positive pleasure in proportion as our powers are exercised, but not over-exercised; we feel positive pain, in proportion as they are compelled either not to operate or to operate too much. All pleasure, thus, arises from the free play of our faculties and capacities; all pain from their compulsory repression or compulsory activity."

In further illustration, Sir W. Hamilton cites in part St. Austin's account of the influence wrought upon his friend Alypius by the spectacle of a combat of gladiators. The story, as told by St. Austin, is of such great interest for our present purpose, that we give it without curtailment:—

"He [Alypius], not forsaking that secular course which his parents had charmed him to pursue, had gone before me to Rome, to study law, and there he was carried away with an incredible eagerness after the shows of gladiators. For being utterly averse to and detesting such spectacles, he was one day by chance met by divers of his acquaintance and fellow-students coming from dinner, and they, with a familiar violence, haled him, vehemently refusing and resisting, into the Amphitheatre during these cruel and deadly shows, he thus protesting:—'Though you take my body to that place, and there sit me, can you force me also to turn my mind or my eyes to those shows? I shall, then, be absent while present, and so shall overcome both you and them.' They hearing this, led him on, nevertheless, desirous, perchance, to try that very thing, whether he could do as he said. When they were come thither, and had taken their places as they could, the whole place kindled with that savage pastime. But he, closing the passages of his eyes, forbade his mind to range abroad after such evils; and would he had stopped his ears also! For in the fight, when one fell, a mighty cry of the whole people striking him strongly, overcome by curiosity, and as if prepared to despise and be superior to it whatsoever it were, even when seen, he opened his eyes, and was stricken a deeper wound in his soul than the other, whom he desired to behold, was in his body; and he felt more miserably than he, upon whose fall that mighty noise was raised, which entered through his ears, and unlocked his eyes, to make way for the striking and beating down of a soul, bold rather than resolute, and the weaker, in that it presumed on itself, which ought to have relied on Thee. For so soon as he saw that blood, he therewith drank down savageness; nor turned away, but fixed his eye, drinking in pleasure unawares, and was delighted with that guilty fight, and intoxicated with the bloody pastime. Nor was he now the man he came, but one of the throng he came unto, yea, a true associate of theirs that brought him thither. Why say more? He beheld, shouted, kindled, carried thence with him the madness which should goad him to return not only with them that first drew him thither, but also before them, yea, and to draw in others."*

Those who are familiar with the horrible scenes of a Spanish bull-fight will, perhaps, most readily appreciate the vigour and truthfulness (if we may so speak) of this description. Even now, at a distance of several years, we can recal vividly the irresistible fascination which, when first we witnessed a bull-fight, overcoming the sickening disgust that had at the onset seized upon us, fixed us to

* *Confessions*, b. vi. c. 8.—Trans. revised by Dr. Pusey.

the seats of the arena. We were hemmed in by a bevy of elegantly attired ladies. The first bull, a magnificent, sharp-horned, clean-limbed, savage brute, had just entered the ring, and, almost without a pause, rushing upon one of the piçadors, had plunged its horn up to the very root into the chest of the animal he bestrode, and hurled horse and rider over as if from a catapult. The voices of the women outvied the men in crying "Bravo, bull!" and heaping pleasant epithets upon the brute, which now stood with head erect, beating its tail upon its flanks, and glancing its glaring eyeballs around, near the centre of the arena. The piçador was hastily dragged from beneath his horse, and the latter struggled to its feet, but almost immediately fell again, the blood pumping out from the ghastly wound in the breast. We could not withdraw our eyes from the horrible sight, and were compelled, as it were, perforce to keep them riveted upon the agonizing convulsions and struggles of the dying animal until it lay a lifeless carcass on the sand. In the meanwhile the scene had been again and again enacted, with variations too fearful to recount, and several horses, some dead, others dying, strewed the arena. At each successful plunge of the maddened bull the excitement of the vast multitude had increased step by step, until it culminated in one frantic tumult of applause, when one horse was literally ripped open from the thigh to the breastbone, and the rider hurled senseless to the ground. Then the welkin was filled with exulting shouts; the amphitheatre trembled beneath the thunderous beatings of the long reeds which many of the spectators held in hand; brave terms were showered upon the gallant bull, and a yell of execration was hurled at the unfortunate piçador as he was raised from the ground and carried from the ring. How we sat the scene out, and much that followed, is a marvel to us now, but we could not drag ourselves from the spot. We were fixed, as it were, by a spell, and it was hard to resist joining in with the rapturous excitement around.

The fascination which the contemplation of murder exercises upon us appears to be, in its sensual aspect, closely allied to, if not in its essence identical in character with, that which seizes hold of the feelings when we gaze upon a spectacle of fierce and bloody contention, such as those to which we have referred in illustration. It is an ultimate fact, which admits of no explanation, and which is linked to that instinctive passion for physical action that is peculiar to all animals, and which is most conspicuously shown in man by his warlike propensities. "Every animal," Adam Ferguson has finely said,* "is made to delight in the exercise of his natural talents and forces: the lion and

* *Essay on the History of Civil Society*, pt. i. sec. 4: quoted by Sir W. Hamilton.

the tiger sport with the paw; the horse delights to commit his mane to the wind, and forgets his pasture to try his speed in the field; the bull, even before his brow is armed, and the lamb, while yet an emblem of innocence, have a disposition *to strike with the forehead,* and anticipate in play the conflicts they are doomed to sustain. Man, too, is disposed to opposition, and to employ the forces of his nature against an equal antagonist; he loves to bring his reason, his eloquence, his courage, even his bodily strength to the proof. His sports are frequently an image of war; sweat and blood are freely expended in play, and fractures or death are often made to terminate the pastime of idleness and festivity. He was not made to live for ever and ever, his love of amusement has opened a way to the grave."

This passion for the free exercise of the physical powers we possess underlies that emotional attraction to which the unrestrained and utmost exhibition of those powers, however sad and revolting, gives rise to. How hard it is to eliminate or keep in check, even under the happiest circumstances, the instinctive delight for violent or dangerous sports, was pretty conclusively shown by the outburst of popular enthusiasm which broke out, in this country, last year, on the brutal combat between Sayers and Heenan.* At the present time, also, M. Blondin's terribly

* While this article was passing through the press, a Prize-fight occurred—a so-called "Fight for the Championship"—between one Mace, a very small man, "Champion of the Light Weights," a professed pugilist, and a gigantic Lancashire blacksmith, named Hurst, of Herculean strength, but unskilled with his fists. The following extract is from the account of the fight in the *Times* (June 19th) and it is worthy of note that the same paper on that day reports that the Secretary of State, the night previous, had announced in the House of Commons, that he had effectively interfered in putting a stop to one at least of M. Blondin's horrible freaks, to wit, his making a little child a party to his dangerous performances. Is it not a curious coincidence that at the time when the Crystal Palace Directors are maturing a plan by which they can evade the law, and open the Palace grounds to a great extent to the public on the Sunday, they should also secure M. Blondin's services for the delectation of the people? The former step is sought to be justified by the ordinary platitudes of the physical welfare of the people, Sunday a day of bodily as well as spiritual rest and enjoyment, and so forth. But in what category of the innocent and instructive amusements, which the Company profess to cater for the public, does M. Blondin rank? The appearance of M. Blondin in England, under such patronage, casts a very dubious light upon any efforts for relaxing somewhat the strictness of Sunday observances under the same patronage.

"Hurst," the report of the fight in the *Times* states, "knew evidently nothing of boxing, and his antagonist therefore merely drew aside with the most perfect *sang froid* from the slow, awkward movements of the ponderous arms, delivering his own strokes full on the head and face of the giant with a force and rapidity that was terrible. In vain, like a blind Cyclops, Hurst threw his arms abroad, and strove to grasp, to strike, even to touch, his lithe wiry foe—in vain he strove to hem him into a corner. Mace would simply inflict his tremendous blows full on the smashed face of his opponent, pass under his arm, and be gone, almost before the eye could follow his movements. Hurst was literally deluged with blood, which poured over his huge figure in such streams that Mace himself was covered with it, and the clothes of Hurst's two seconds almost saturated. Nothing showed the enormous strength exciting and dangerous gymnastics at the Crystal Palace are calling forth another phase of the same feeling—heightened, doubtless, by the fact that a fellow-acrobat was crippled for life by exhibiting less dangerous feats in town a few weeks ago.*

But there is a psychical as well as a sensual phase of this passion for the terrible. In the latter, the more instinctive phase, man is barely elevated above the level of the brute beast. There is no stronger evidence of an individual becoming or being *brutalized* than the sensual phase of the passion assuming in him an irresistible sway. It is only when we turn to the psychical phase that we recognise the true significance of the passion in man, and in what manner emotions seemingly inconsistent with true humanity become the substratum and vital spring of the highest humanity. For, if on the sensual side these emotions are characterized by a mere brutal gratification in the horrible, on the psychical, they

---

of the man more than that he could sustain this fearful punishment and loss of blood with apparently little diminution of his colossal power. He still pursued Mace with unabated determination, but never once even touched him, while, on the other hand, Mace's blows sounded loud all over the ring, till from a sharp crushing smash they gradually deadened down to a splashing sound like striking raw meat, that was sickening to hear. Nothing stopped the copious streams of blood that flowed from all parts of Hurst's face, and the whole of this one-sided contest became disgusting and horrible beyond all description. After there had been ten rounds, and the fight had lasted some three-quarters of an hour, Hurst's seconds and backers saw that his chance was hopeless, and urgently strove to make him discontinue. But, though now utterly blind, his features smashed out of all recognition almost as a human being, and reeling from his fearful loss of blood, the gory disfigured giant still tottered from his corner, only to be sent staggering back by an antagonist that he seemed capable of annihilating. Mace now no longer fought cautiously, but hit when and where he pleased, and even closed with the great wrestler and threw him heavily. It was all over. Hercules himself would have succumbed to such fearful blows, and the alarming hæmorrhage which followed them, and which now began to soak all the grass of the ring. Brettle, Hurst's chief backer, at last rushed into the arena, and insisted on his fighting no more, but the maimed giant seemed incapable of understanding his defeat from such a little man, and groped and staggered out again. Blind and fainting, it only required one or two more blows to finish the affair; but the infliction of those on the helpless heap of flesh was horrible and sickening beyond all description. His seconds and backers gave in for him without his knowledge, and kept Hurst in his corner till he gradually became almost insensible, and all the restorative arts of the ring were exhausted in efforts to keep him from fainting, which, in the absence of a surgeon, and in his then fast failing power, might have been a most serious affair. The spectacle which he presented is too horrible for description. Even the oldest champions of the ring were aghast at the fearful punishment inflicted in fifty minutes. Mace had not a single mark on him. The dockyard police were despatched in a steamer from Chatham to prevent the fight, and arrived just as it was over—quite official that. It is a kind of set-off to this revolting business to say that poor Hurst's comrades on both sides were most solicitous in their care of him after his defeat, and Mace went about among them and raised a subscription for him amounting to 35*l.* Such facts, though undoubtedly praiseworthy, but poorly counterbalance the nature of the whole contest. Yet pugilists think that in a few years the ring will again glow with all the brutal magnificence of the days of the Regency. Revive the ring! It would be easier to restore the Heptarchy."

* In the arena where the accident happened, a still more daring acrobat is now nightly delighting a crowded audience.

arouse and minister to the holier feelings of sympathy and pity, and are the most powerful awakeners of the idea of justice. It is this then, the psychical phase, and the capacity of manifesting it, which constitutes the distinctive and true character of this passion for the horrible in man. The perfecting of this capacity rests with man himself.

We may now comprehend in what fashion the fascination which murder exercises over us contributes to the higher feelings of humanity. If the act simply affected us with blank horror, and there was no recoil of the feelings, neither sympathy, nor pity, nor justice would be practicable. Every energy of the mind would flag and fail whenever and however the act were contemplated, and we should seek at all hazards to avoid the terrible theme. But by an inscrutable law of nature the horrible itself has its peculiar attractions; we are drawn instinctively to its contemplation; and from that contemplation arises, in the rightly-constituted mind, emotions that dignify and ennoble man. Thus, as the least consequence, murder sets every mind on the stretch, and the murderer is tracked not merely by the officers of the law, but by the active thoughts of an entire people.

But the psychical phase of the passion for the terrible is not only manifested through the ethical feelings, but also through the æsthetical. "Whatever is in any sort terrible," writes Burke,† "or is conversant about terrible objects, or operates in a manner analogous to terror, is a source of the sublime." The tragic poet seeks in murder the materials upon which to exercise his great art; and the Tragic Muse is depicted with the dagger in one hand, the poison bowl in the other. As Bulwer Lytton has happily said, Crime "has afforded to the master of human nature his amplest scope for investigating the most subtle and hidden recesses of character and passion, unravelling the skein of intellectual error, and holding up to a thoughtless world those striking and solemn warnings in which the most direct morality of tragic composition may be said to consist."†

De Quincey, in his extravagant essay on "Murder considered as one of the Fine Arts," keenly satirized a perverted or debased taste in the contemplation of the act, which is occasionally met with, and which is best left to the lash of the satirist.

Of the causes which contribute to the energy of the emotions with which we regard murder, and which most intensify them, mystery and strangeness occupy the chief place. The latter provokes to fuller action the most dominant feelings, the former prevents these flagging. But the action of both causes is to a

---

* *On the Sublime and Beautiful,* part i. sect. 7.

† A Word to the Public: Appendix to *Lucretia.* Ed. 1853.

great extent relative to other circumstances, which are liable to a wide range of variation in degree of intensity, and which are intimately concerned in fostering and augmenting the horror with which murder affects us. These circumstances may be summed up in the term *domesticity*, by which we mean to convey the notion of domestic or family life in its widest sense. For example, in the instance of the murder recently discovered on the Rhine, and to which the attention of the English public has been so forcibly directed by successive advertisements in the *Times*,* mystery and strangeness exist to an extent rarely paralleled, yet despite the interest with which we regard the event, we miss the *swell* of emotion with which murder of a character so barbarous commonly affects us. No doubt, in this case, something must be ascribed to distance in weakening the influence which the crime exercises over us. But, even in the perhaps still more striking example, the discovery of murdered and butchered remains on Waterloo-bridge several years ago, notwithstanding the horrible character of the crime and its impenetrable mystery, we question if for a moment it would be maintained that the feel-

---

* *Advertisement from the* TIMES *of May* 9, 1861.

"In an almost impenetrable ravine in the declivity of Mount Rheineck, which is situated immediately on the banks of the Rhine, between Brohl and Niederbreisig (a district of the Tribunal of First Instance of Coblentz, Rhenish-Prussia), on the 22nd of last March, was found the body of some person, a female, from twenty to thirty years of age, or thereabouts, concealed in a recess, covered with large stones. The period of decease cannot be precisely determined. Death was caused by a ball shot from a gun, which traversed the breast and back. Description—height, five feet two or three inches; hair, fair; teeth, sound, small, and somewhat irregularly set in the lower jaw. Dress—one chemise, cambric, three feet six inches long, the upper hem forming a running string, with two eylet-holes, two fine and even cords passing through; in the centre of the round breast of the chemise, and below the eylet-holes, the initials 'A. H., 36,' are embroidered in Gothic characters, in relief, half an inch long. 2. A night-gown of fine white dimity, collar turned down, two feet three inches, with white mother-of-pearl buttons; some remains of a fine material, with brown and white stripes (jaconot muslin); in the white stripe is a small winding white line, with red spots. In the vicinity of the body have been found the remnants of a petticoat, three feet two inches long; it is composed of fine white dimity, striped, the same material as the nightgown. On the upper edge, which is an inch and a half broad, with white riband strings, are embroidered in white letters, two and a half lines, in relief, and in large characters of the German printed alphabet, the initials 'M. R., 6.' The bottom hem is finished with a cord in linen thread. The fine quality of the materials and the elegant make of all these articles indicate that the victim belonged to a rich class. In consequence of the state of putrefaction and external destruction it is impossible to notice other marks of recognition. I request of any person who can give information concerning this unknown individual, and the circumstances of her death, to be so good as to furnish me with the particulars, else to communicate them to the nearest magistrates. The articles of dress above mentioned, together with the lower jaw, are deposited for inspection at my office.

"The Crown Prosecutor-General,

"DE RODENBERG.

"*Coblentz, April* 25, 1861."

ings excited by it at all approximated in intensity or duration to those aroused by the murder at Road Hill House, twelve months ago.

The influence of *domesticity* in heightening the emotions excited by violent death admits of ready illustration.

It has been our lot to become exceedingly familiar with Death in almost every form—in his quieter and more seductive, in his harsher and more repulsive moods—as he has arrested life in bed, in the highways and byways of the country, in the crowded streets of the town—now when he has suddenly tripped up an individual as he was going soberly about his daily avocations or was rollicking in the mad glee of a drinking-bout, now when he has swept along in the pestilence or held high carnival on the field of battle. For months our chief associates have been the dead. We have lived among them from early morning until midnight, and often from midnight until daybreak; and when we sought rest, still but a thin wall of lath and plaster separated us from them. The suicide, the gallows-bird, and the "found dead," the vagrant, the deserted, the lost—such were our lifeless and lone companions by night; and frequently as midnight has stolen into morning, have we sat among them, and by the light of a lamp, or by the pale rays of the moon, we have gazed long upon the hushed forms of our ghastly and undraped comrades, listening to the wind as it whispered about the windows, and at times watching curiously the sudden motion of a straggling lock of hair, which, tossed up by a stray eddy, would give a temporary semblance of vitality to a corpse. A singular kind of friendship, the result of our solitary vigils, sprang up between ourselves and the dead; but notwithstanding the extreme familiarity thus created with death, it never blunted that strange thrill of fear which death as Murder communicated to us. Even when we were living, so to speak, hand and glove with the dead, we could not entirely overcome the dislike to pass, by night, along the lane where the pedlar was found beaten to death, or the copse where the missing gamekeeper had been discovered shot through and through and drained of his life's blood, or the deserted cottage where the assassin's knife not long ago was too busily at work, or the foliage-fringed pond from which the body of the little infant had been fished out. But this by the way.

It is a strange study to mark the influence of death upon the feelings as met with on the field of battle, in the deadly routine of the trenches, under the horrible mitraille of a close cannonade, or in the fierce rush and hideous confusion of the assault, the mine springing like a volcano beneath the feet of the men in advance, and rending them to pieces amid fierce tongues of vivid flame.

— It is a clear hot day in the early spring of 1855. A

brisk breeze is crisping the dark blue waters of the Euxine, and Sebastopol, lying like a white cloud upon the deeply indented shore, looks the very emblem of peace as we gaze upon the city from the ridges of the hills that hem it in. But for the rushing out of jets of pure white smoke from the earth, as it were, at intervals, along the slopes that run down to the defences, and the loud reverberation of a heavy gun from time to time, and the occasional sudden flecking of the pure sky above the devoted city with a bursting shell, there is little at first to tell of war. Let us approach nearer. We have strayed into Gordon's Battery. The ponderous guns are comparatively idle, and the men are lounging beneath the vast earth-works. Presently the Malakhoff and Redan awake up and hurl a storm of round shot and shell into the battery. Keep close! A slight report in the air above us, a sharp whiz, a jagged, black fragment traverses the field of vision, and in a moment the tall, fine sailor to the right is torn open, a quiver of the limbs, and he is dead. A bustle around the body, a hasty exclamation or two of regret—but, keep close! Round shot and shell are plunging into and tearing up the ground in the rear of the guns. A piece of canvas is thrown over the body until it can be removed, and Jack quietly turns again to do the honours of the battery to the startled stranger, and even ventures on a joke or two. Is this insensibility?

— The Mamelon has fallen, and a truce has been declared for the burial of the dead. Eighty corpses, horrid in the hideous disfigurement of wounds and approaching putrefaction, have already been laid in two rows in rear of the second parallel, preparatory to burial. The trenches in the vicinity of the dead are crowded with men, who, while watching the movements of the burial parties, fret the tainted air with laughter, and jest, and oaths. Shut the eyes, and you might think yourself in a crowded cabaret; open them, and they rest on the two lines of dead, who, with faces turned towards heaven, lie before their mirth-brimming comrades, with whom they had fought shoulder to shoulder but two nights ago.

— The plain of the Tchernaya, rich with luxuriant grass and gay with flowers, is flooded with morning sunlight. A long line of dust beneath the distant cliffs marks the track of the retiring Russian columns, but an occasional shot is still thrown, from the heavy guns at the foot of the hills on the left, upon Tractir Bridge, around and about which the dead and the dying are lying in profusion. Mind your horse does not strike the wounded as you push down to the bridge, or become entangled with the dead as you ford the river. But draw rein as you reach the opposite bank. What strange sound is this? The cry of the wounded, hundreds of whom are lying within the glance of the eye, and hundreds more are concealed in the long grass? No! simply full-voiced laughter, overbearing even the brisk talk of the victorious troops on the bridge and within the breast-work to the right and left of it. Turn aside a little, and in a slight hollow, fringed sparsely by brushwood, we come upon half-a dozen Russian soldiers, all so severely wounded that not one can stand erect. They have had their wounds dressed on the field, and are awaiting their turn for removal, and in the meantime they are making merry with the French soldiers in the immediate vicinity. They shout out harsh sentences, doubtless rough Russian jests if they were understood, and then burst into paroxysms of laughter, in which their captors are not backward in joining. A merrier group could not be desired, yet it is fixed in the midst of a very crush of the dying and the dead. Within a little mile 3000 of the latter were lying.

— Once more. It is early morninig after the fall of the Malakhoff. The passages between the gigantic walls of earth are cumbered with the dead and wounded. Here and there corpses are piled upon corpses, and the flaunting red and blue of the French uniform peers out beneath the brown of the Russian. It is but needful to glance briefly around to learn in how ghastly yet how varied a guise, shell, and shot, and bayonet, and powder, and fire can do their work. It is a very saturnalia of the dead and dying; and in sullen yet bootless vengeance a shell is ever and anon thrown into the midst of it from the Russian batteries across the harbour. Yet the sounds we hear would befit a saturnalia of the living rather than dead. There is a pile of (we counted thirty) bodies, French and Russians, and near it are squatted a group of soldiers, surrounded by plunder, and singing with the fullest voice a jovial drinking-song. The shattered defences ring to the song, and the chorus itself is shouted back by many voices in other parts of the conquered work.

And so we might multiply almost endlessly fact upon fact, showing how little violent death, however horrible, in a foughten field, away from any of the incidents of home, affects the feelings of the combatants. Yet we also might multiply other instances, showing that this arises from no want of sensibility. From the Malakhoff, from the Mamelon, from the Tchernaya, we could cull stories of overbrimming kindness towards friend and foe, of tenderness towards the wounded more like that of a woman than of a fierce and hardy man, and of kindnesses towards the dying which make the heart beat with emotion. To the dead there is no help—to the living there is; and the soldier consigns his comrade hastily to the grave, with a brief expression of regret, knowing that he himself may be laid in one to-morrow, and reserves his active sympathy for the living. But the same man who in war seems to be heedless as a brute, or, very like a brute, appears, as it were, to revel in blood and death, in civil life might be utterly unnerved to witness a man accidentally killed or seriously injured, and might dread to be brought face to face with death in a sick-bed. We have known veterans of many a bloody fight sicken to see a child's crushed finger, and fear to sleep in the same house with a corpse.

Whence comes this marvellous difference of feeling in the same man in contemplating Death? The answer is not far to seek, and is to be found in the absence or slight degree of home feeling which surrounds death in the one case, its presence in the other. Death under any circumstances excites a nameless fear, but the tension of this fear is in direct proportion as death intrudes upon the sacred hearth of domestic life. It is a fear that is most acutely experienced among home-born joys, that is fullest fed by the wailings of children, of parents, of friends, of all who are dearest in consanguinity or amity—wailings which surround the death-bed with an atmosphere of grief, from the influence of which none can escape—a grief which augments in intensity with the abruptness of the dread intrusion. Death comes in its mildest form in sickness, for we all expect it, and know that sooner or later it is most likely to come, in this form; hence, when it approaches thus, even when sudden like a thunderbolt, we are less shocked than when it comes upon us by a sudden accident. But even death by an accident—say by the destruction of a railway-train—is a form of its visitation to which, as we are compelled to admit, we are liable in a very appreciable degree, and thus a certain amount of submission qualifies the terrible event. But death by MURDER!—we cannot, or rather we will not, estimate any chance, any likelihood in the ordinary life of a civilized country, of its drawing near to us in this fashion, a fashion which passes beyond the reach of common probabilities. And so when it thus does come upon us it comes with a nakedness of horror which puts to rout even the laxest morality, and stirs up the most sluggish soul from its deepest foundation. Our sense of security from death, in short, is greatest in respect of murder, less in prospect of accident, and least of all in face of sickness; and the shock to the feelings occasioned by death is in no small degree proportionate to the disturbance of the degree of security we ourselves feel from its approaches. Add the element of domesticity—of home feeling, of family life—and we may, in some sort, measure the variation in degree of effect with which even murder, horrible at all times, but even itself susceptible of increment of horror, affects us. Thus we may learn to apprehend, in some respects at least, how it came to pass that the murder of Mrs. Ruscombe and her maid, in the middle of last century, of the Marr family, at the beginning of this, and of Mr. Kent's child at Road, last year, exercised so awful and enduring an influence upon the people—three examples of murder which (to use an expression of Southey in respect to the destruction of the Marr family) were private events of that order which rose to the dignity of national events.

Mrs. Ruscombe lived with a single maid servant in College Green, Bristol. One morning they were both seen alive, and moving about, as usual, at ten o'clock; at noon both were found murdered. The mistress lay dead in her bedroom; the servant upon the stairs. To this day not one ray of light has been thrown upon this strange murder, perpetrated near midday, in a house overlooking a busy street, and in the heart of a busy city.

The Marr family lived in the vicinity of Ratcliffe Highway. Marr kept a small shop, and late one Saturday evening, in 1812, while the servant girl was out on an errand, he, his wife, the apprentice boy, and infant child, were all deliberately murdered. Marr was found dead behind the counter, the wife and boy in the centre of the shop-floor, and the baby in its cradle in the kitchen. The murderer had escaped through a yard in the rear of the house, but for the rest all was mystery. A fortnight after the occurrence of this terrible series of murders, another house was entered in the same vicinity, also on a Saturday evening late, and the head of the house, an old man of seventy, named Williamson, his wife, and servant-maid killed whilst pursuing the avocations they were engaged in at the time. It was manifest that these murders were effected by the same hand or hands that had extirpated the Marr family, and the awful and wide-spread fear excited by the previous murders increased to a literal panic when the murder of the Williamsons had been discovered—a panic which hardly ceased when the murderer (Williams) had been happily discovered and brought to justice. Speaking of this panic to De Quincey, Coleridge observed that he did not consider it at all unreasonable, "for in the vast metropolis there are many thousands of households composed exclusively of women and children; many other thousands there are who necessarily confide their safety, in the long evenings, to the discretion of a young servant girl, and if she suffers herself to be beguiled by the pretence of a message from her mother, sister, or sweetheart into opening the door, there, in one second of time, goes to rack the security of the house."*

* "It would be absolutely impossible adequately to describe the frenzy of feelings which," writes De Quincey, "throughout the next fortnight (after the Marr's murder), mastered the popular heart; the mere delirium of indignant horror in some, the mere delirium of panic in others. For twelve succeeding days, under some groundless notion that the unknown murdered had quitted London, the panic which had convulsed the mighty metropolis diffused itself over the island. I was myself at that time nearly three hundred miles from London; but there and everywhere the panic was indescribable. One lady, my next neighbour, whom personally I knew, living at the moment, during the absence of her husband, with a few servants in a very solitary house, never rested until she had placed eighteen doors

Equally great had been the panic excited by Mrs. Ruscombe's murder, but, if aught, it proved more lasting from the impenetrable mystery which enshrouded it.* Both cases begot a terrible sense of domestic insecurity, that, unfortunately, we are but too well enabled to appreciate from the recent experience of the Road murder, which in barbarity and in mystery exceeds even that of Mrs. Ruscombe and maid. But in all these three cases we may see that it was not so much the mystery, temporary or persistent, the strangeness, or the barbarity of the crime which constituted the chief elements of panic, but the *domesticity*, and the awful feeling of insecurity carried in consequence into every household. What family was or could be safe from the intrusion of murder under such guises? Not one.

No incident, De Quincey tells us, referring to the murder of the baby in the massacre of the Marr family—no incident, throughout the whole tissue of atrocities, so much envenomed the popular fury against the unknown ruffian, as the useless butchery of the infant. Nothing, it may also be said, aided more in intensifying popular feeling in the Road Murder than the fact that the victim was an infant. "It gives me the horrors to think of it," said one of the witnesses on the trial of the nurse-girl in that case; "I'm the mother of a large family myself, and know it." The child has a deeper hold upon our feelings than the adult. The helplessness and entire dependence of the former binds it to us with stronger-welded and purer links than those which attach man to man. How often and how painfully does the doctor experience this at the bedside. From his older patients he learns all he may want to know, and in them finds intelligent and willing assistants in all that he may wish to do; but not so with the infant. It can resolve no doubt, clear up no difficulty. It is entirely passive in his hands; entirely dependent upon his unaided skill and acuteness for relief of its sufferings. "Help me," the little thing seems to say by its cry, "for I am utterly helpless without you." And not only is the doctor's responsibility thus doubled, but his child patients contrive, in a thousand winning fashions, to creep into his heart, and

---

(as she told me, and, indeed, satisfied me by ocular proof), each secured by ponderous bolts, and bars, and chains, between her own bedroom and any intruder of human build. To reach her, even in her drawing-room, was like going, as a flag of truce, into a beleaguered fortress; at every sixth step one was stopped by a sort of portcullis. The panic was not confined to the rich; women in the humblest ranks more than once died upon the spot, from the shock attending some suspicious attempts at intrusion upon the part of vagrants, meditating probably nothing worse than robbery, but whom the poor women, misled by the London newspapers, had fancied to be the dreadful London murderer."

* Fifty years after the murder, the house in which it was effected remained deserted.

seize his affections, and so again to deepen still more his anxieties about them.

The Road Murder has recently been invested with additional interest by the publication, in a handsomely printed volume, with plans and illustrations, of the whole of the judicial evidence relating to it, and a critical review of that evidence, by Mr. J. W. Stapleton, surgeon, of Trowbridge, Wilts. Mr. Stapleton was present at the examination of the murdered infant, although not himself examined as a witness at any time. In other respects, also, he was placed in a favourable position for watching the progress of the case. His opinion upon the mode of murder differs somewhat from that given in evidence by the surgeon who officially examined the body, Mr. Parsons, and his criticism clears up one or two doubtful points in the case. But the great value of Mr. Stapleton's work consists in its presenting a connected and trustworthy account of one of the most remarkable crimes in English domestic history, and for this reason it will always prove of unusual interest.

The actual facts of the Road Murder are few and quickly summed up. On the morning of the 30th of June, 1860, the youngest child of Mr. S. S. Kent, an Inspector of Factories, residing at Road-hill House, Wiltshire, a fine healthy boy, four years of age, was found murdered, in a disused privy adjoining the house. The body, clad in a night-dress and under-flannel, and wrapped in a blanket from the cot in which the child slept, had been thrust down one of the apertures of the seat in the privy, but had been prevented falling into the soil beneath by the interposition of a splash-board. The throat was cut clean down to the spine, and a deep stab penetrated the chest on the left side. There was also a slight cut upon the fingers of the left hand. Much blood was found upon the inner side of the blanket, and some had trickled through and fallen into the soil beneath where the body of the child lay. Mr. Parsons considered that the child had been suffocated before the wounds were inflicted; Mr. Stapleton believes that the wounds were alone the cause of death; and in this view he is supported by the opinion of the coroner who held an inquest on the case, also a medical gentleman. It has generally been understood that the quantity of blood found in the vicinity of the body was much less than might have been expected, where death had arisen from extensive wounds, particularly when large bloodvessels had been divided; but Mr. Stapleton shows that this opinion is hardly tenable, as Mr. Superintendent Foley, of the local police force, has testified that the water in the vault beneath the body was stained, as he thought, "with much blood." A piece of flannel, described as a female's breast-flannel, was found beneath the body, and a fragment of the *Times* newspaper of June 9th, lay on the floor of the outhouse. This piece of paper had apparently been used to wipe a bloody knife; but it was impossible to determine whether the breast-flannel had been thrust into the position in which it was discovered along with the child, or whether it had fallen into the vault some time previously. Moreover, no trace of the previous ownership of the flannel could be discovered.

For the rest; on the evening previous to the discovery of the murder, Mr. Kent's family, consisting of twelve members, including servants, had retired to rest as usual, Mr. Kent seeing that the house was safely secured for the night. The murdered child slept in a cot in the night nursery, another and younger child occupying another cot, and the nurse a bed in the same room. Early in the morning the nurse awoke, and noticed that the little boy's cot was empty; but, according to her statement, supposing that Mrs. Kent might have removed the child from the room, having perhaps heard him cry in the night, she went asleep again. She once more awoke about half-past six, and arose; but the fact of the child not being in Mrs. Kent's bedroom was not discovered by her until the usual time of her master and mistress rising, when she knocked at their bedroom door and asked for the child. She stated that she had tapped at the door once before, but no answer being given, she went about her usual duties until the ordinary time of Mr. Kent's dressing.

The housemaid, on first coming below stairs that morning, had been surprised to find the drawing-room door unlocked and slightly a-jar, and the shutters of one of the windows open, the window itself being slightly raised up. She, however, thought that the window might have been opened by some member of the family the previous night, to cool the room, after the servants had gone to bed, and accidentally left open, together with the door, and therefore she raised no alarm.

The child being missed, a search was at once instituted for it, and after some lapse of time the body was found as described. No sign of any one having forced an entrance into the house was discovered, and the only indications of egress from it were the open drawing-room door and window. The cot from which the child had been removed was not tumbled in the least, and the cot-sheets were found neatly turned back, as if by a practised hand, a blanket having been abstracted from between them. Not the slightest trace of anything connected with the murder could be detected within or without the house, except in the closet where the child was found, as we have already described. There could be no doubt, however, that the murderer must have been secreted within, or an inmate of, the house; but beyond this conclusion, the keenest investigation was at fault. The suspicions of the police first fell upon one of Mr. Kent's daughters, a young lady sixteen years of age. Some asserted expressions of dislike towards the murdered child, and a missing night-dress belonging to the young lady, and which could not be accounted for, seemingly gave some colour to the suspicions, and she was arrested. Nothing, however, was brought forward in evidence before the magistrates to justify in the least degree her detention. Subsequently the nurse girl was arrested on suspicion, but in her case also there was not a tittle of evidence to connect her with the perpetration of the deed. The police were, in fact, utterly baffled; and the public, angered at the failure of justice, spared neither the authorities concerned in the investigation of the crime, nor Mr. Kent's family, but condemned the efforts which had been made by the one, and indulged in the gravest suspicions against the other. This outburst of popular feeling, notwithstanding the reprehensible character of its results in some respects, was perfectly natural. For the nation was called upon to rest content (and must unhappily perforce submit to do so) with the bare facts that, in an English home, on a summer's morning, in June, 1860, a lovely child, sleeping in the same room with its nurse, was taken out of its cot, removed from the house without any one of several sleeping inmates being disturbed, and murdered without the slightest trace of the murderer being discoverable. Strange indeed it would have been had public .feeling remained passive under such circumstances. Doubtless there have been murders as mysterious, murders as anomalous, murders infinitely more barbarous in details, but never a murder in which mystery, singularity, and barbarity were so calculated to arouse whatever feelings of horror attach to the crime—never a murder so calculated to awaken a deep sense of family insecurity—never a murder so diabolical in its unoffending and innocent object, and in the exquisite care with which it was effected and concealed under difficulties of no ordinary character, or so astounding in the inexplicability of its motive. Whatever, indeed, could have contributed to the intensity of our emotions in the contemplation of murder (if the previous indications given of the circumstances governing those emotions are worth aught) is found present in the case of the Road Murder.

Having regard to this case, and looking back to the murder of Mrs. Ruscombe and maid, the Marr family and the Williamsons, not forgetting the more recent deeds of Good, the Mannings, Bacon, Rush, Palmer, Youngman, and Mullins, we are compelled to admit that De Quincey's phrase, applied to the present epoch, *The Augustan Age of Murder*, is not without a deep significance. The Road Murder, indeed, would seem to clinch his uncomfortable proposition (upon which the designation is founded) that more skill has been dis played in effecting murder in the last two centuries and a half than in any previous period of history. We may, however, if we please, flatter ourselves that murder is less common, and even on the whole less barbarous—that is to say, that the barbarity of murder in the present day, for example, is, generally speaking, more refined than in any prior time.

We admit the proposition, and it will presently be seen that one or two somewhat important conclusions flow from it. Since the progress and mutations of crime in this country have been submitted to statistical observation, an opinion which had previously been entertained, to wit, that skill was becoming more and more conspicuous in the execution of crime to the displacement of violence, has been to a certain extent confirmed. Thus Mr. Samuel Redgrave, in his report upon the "Judicial Statistics for 1859," published by the Home Office, states that—"The whole tendency of crime has been, for some years, to the diminution of offences of violence, and the increase of offences of planned theft and fraud—skill in crime has succeeded violence."* It would seem, then, that what has been observed of murder is true also of crime generally, and that the greater prevalence of skilled murder is but a particular instance of a more general phenomenon.

Not unnaturally the more frequent manifestations of skill in crime have given, and give rise to serious surmises as to the probable effects of this greater aptitude for nefarious acts exhibited among the criminal classes. If crime becomes more closely allied to skill, it would seem apparent that its evil influence may be illimitably extended. If murder becomes conspicuous for and is a question of dexterity, and this dexterity be such as to set the best trained police at defiance, who would be safe? Nay, more; does not the frequent repetition of ghastly crimes—of frightful murders—within the last three or four years, indicate that there is not an inverse relation between the frequency of crime and the skilfulness with which it is perpetrated, as some would hope, but that the one increases progressively with the other?

The answer to these doubts is happily furnished to some extent by the statistics of crime we possess. And, first, it is highly probable that there has been no increase in the amount of murder in this country within the last thirty years. This conclusion is founded upon the commitments for murder for that period,† no statistics of the

* *Introductory and Explanatory Report*, p. xvi.

† *Commitments for Murder in Quinquennial Periods, from* 1830 *to* 1859.

| | | | |
|---|---|---|---|
| 1830-34 | 326 | 1845-49 | 365 |
| 1835-39 | 315 | 1850-54 | 348 |
| 1840-44 | 347 | 1855-59 | 345 |

In the thirty years population cannot be estimated to have increased less than 40 per cent. (which would account for a corresponding increase of crime), and property probably in a much greater ratio.—*Judicial Statistics*, 1859, p. xvi.

actual number of murders perpetrated existing until the year 1856. It is curious, however, to remark that there has been a progressive increase in the commitments for attempts to murder, stabbing, wounding, &c., within the same period. "This increase," we learn from Mr. Redgrave, "showed itself in a marked degree in 1857, on the extensive abolition of capital punishments which was then effected."* The amount of murder, therefore, remaining nearly stationary, notwithstanding a relaxation of the laws, followed by an increase of crime attended with serious bodily injury, it may justly be assumed that the more notorious murders of late years are not indications of an excess or increase of murder in the kingdom.

Again, it is not improbable that a greater prevalence of skilled murder (if we may so speak) is itself an index of a state of things much more amenable to restraint, than the prevalence of murder arising from the mere outbursts of animal passion. It may be surmised that the display of an increased skilfulness in crime arises chiefly from the greater difficulties interposed to the perpetration of crime by an active police. Now, Dr. Jarvis, the President of the American Statistical Association, in a paper read before the International Statistical Congress, held last year in London, pointed out that the tendency to repetition of crime was greatest in crimes arising from the passions and feelings, least in those which required or were governed by the intellect. "Passion and appetite being inherent or excited in the animal constitution, or permitted to govern the individual to whom they belong, are not so easily nor so commonly repressed by the ordinary punishments, or restrained by repentance and reason, as the misplaced selfishness which endeavours to succeed by plan and calculation."† This conclusion being confirmed, it is not perhaps unreasonable to infer that, in the case of murder, the restraining influence of the law will prove more effectual in proportion as the influence of passion is curbed and controlled by craftiness and forethought.

Thus a little hopefulness for the future may be extracted even from the murder at Road-hill House; and this hopefulness is strengthened by the fact (vouched for by Dr. Farr‡) that few countries present so low a proportion of murders as England.

---

* *Judicial Statistics*, 1859, p. xvi.

† Dr. Jarvis's paper will be found on p. 391 of the present number of this Journal.—Ed.

‡ *Nineteenth Annual Report of the Registrar-General*, p. 198.

